# Hybrid model of CT-fractional flow reserve, pericoronary fat attenuation index and radiomics for predicting the progression of WMH: a dual-center pilot study

**DOI:** 10.3389/fcvm.2023.1282768

**Published:** 2023-12-19

**Authors:** Jie Hou, Hui Jin, Yongsheng Zhang, Yuyun Xu, Feng Cui, Xue Qin, Lu Han, Zhongyu Yuan, Guangying Zheng, Jiaxuan Peng, Zhenyu Shu, Xiangyang Gong

**Affiliations:** ^1^ Rehabilitation Medicine Center, Department of Radiology, Zhejiang Provincial People's Hospital, Affiliated People's Hospital, Hangzhou Medical College, Hangzhou, Zhejiang, China; ^2^ Jinzhou Medical University, Jinzhou, Liaoning, China; ^3^ Bengbu Medical College, Bengbu, Anhui, China; ^4^ The Hangzhou TCM Hospital (Affiliated Zhejiang Chinese Medical University), Hangzhou, Zhejiang, China

**Keywords:** CT-fractional flow reserve, pericoronary fat attenuation index, white matter hyperintensity, radiomics, machine learning

## Abstract

**Objective:**

To develop and validate a hybrid model incorporating CT-fractional flow reserve (CT-FFR), pericoronary fat attenuation index (pFAI), and radiomics signatures for predicting progression of white matter hyperintensity (WMH).

**Methods:**

A total of 226 patients who received coronary computer tomography angiography (CCTA) and brain magnetic resonance imaging from two hospitals were divided into a training set (*n* = 116), an internal validation set (*n* = 30), and an external validation set (*n* = 80). Patients who experienced progression of WMH were identified from subsequent MRI results. We calculated CT-FFR and pFAI from CCTA images using semi-automated software, and segmented the pericoronary adipose tissue (PCAT) and myocardial ROI. A total of 1,073 features were extracted from each ROI, and were then refined by Elastic Net Regression. Firstly, different machine learning algorithms (Logistic Regression [LR], Support Vector Machine [SVM], Random Forest [RF], k-nearest neighbor [KNN] and eXtreme Gradient Gradient Boosting Machine [XGBoost]) were used to evaluate the effectiveness of radiomics signatures for predicting WMH progression. Then, the optimal machine learning algorithm was used to compare the predictive performance of individual and hybrid models based on independent risk factors of WMH progression. Receiver operating characteristic (ROC) curve analysis, calibration and decision curve analysis were used to evaluate predictive performance and clinical value of the different models.

**Results:**

CT-FFR, pFAI, and radiomics signatures were independent predictors of WMH progression. Based on the machine learning algorithms, the PCAT signatures led to slightly better predictions than the myocardial signatures and showed the highest AUC value in the XGBoost algorithm for predicting WMH progression (AUC: 0.731 [95% CI: 0.603–0.838] vs.0.711 [95% CI: 0.584–0.822]). In addition, pFAI provided better predictions than CT-FFR (AUC: 0.762 [95% CI: 0.651–0.863] vs. 0.682 [95% CI: 0.547–0.799]). A hybrid model that combined CT-FFR, pFAI, and two radiomics signatures provided the best predictions of WMH progression [AUC: 0.893 (95%CI: 0.815–0.956)].

**Conclusion:**

pFAI was more effective than CT-FFR, and PCAT signatures were more effective than myocardial signatures in predicting WMH progression. A hybrid model that combines pFAI, CT-FFR, and two radiomics signatures has potential use for identifying WMH progression.

## Introduction

White matter hyperintensity (WMH) is a neuroimaging feature in magnetic resonance imaging (MRI) that indicates small vascular lesions in the brain. The specific manifestation is signal hyperintensity in the periventricular or deep periventricular white matter on the T2-weighted or fluid-attenuated inversion recovery (FLAIR) images (1). Several previous studies concluded that WMH was associated with cognitive decline, depression, stroke, and even death (2, 3). In addition, the WMH volume may increase or decrease over time (4, 5). Accordingly, early identification of WMH and prediction of progression are essential for preventing the underlying diseases (6). Cardiovascular diseases may provide a pathophysiological background for several brain diseases, such as stroke (7), dementia (8), and cognitive impairment (9). Among them, coronary artery disease (CAD) is closely associated with cerebral white matter disease, but the specific mechanism responsible for their co-occurrence is still unclear (10). The heart and brain have blood vessels with similar anatomical structures, and each organ provides perfusion to tissues through a vascular network of arteries that run on the organ surface (11). Therefore, it is crucial to have a comprehensive understanding of the relationship between WMH and CAD (12).

Previous studies found significant associations of the presence and severity of WMH with cardiovascular health and age (12, 13). The incidence rate of WMH increases with age, and is also affected by cardiovascular risk factors (14). Other studies showed that the presence and a larger volume of coronary artery plaque were associated with a larger WMH volume (10). Coronary computed tomography angiography (CCTA) is a non-invasive method that can provide information on the characteristics of plaque and the severity of lumen stenosis, which was valuable in CAD detection. Vascular stiffness, atherosclerosis, calcification score, calcification plaque and cardiac blood perfusion all have certain influence factors on the occurrence and progress of cerebrovascular related diseases, especially the most common manifestation of cerebrovascular diseases—WMH (15). We can not only directly observe the related manifestations of cardiovascular diseases on CCTA images, but also use some derived markers to further analyze the correlation of WMH through the response to cardiovascular related risk factors. There are currently two CCTA-derived markers that can effectively indicate CAD: the percoronary fat attenuation index (pFAI), which reflects coronary inflammation, and the CT-derived fractional flow reserve (CT-FFR), which reflects hemodynamics (16). The pFAI represents the average attenuation index within the range of PCAT (range of−190 to−30 HU), which is not affected by calcified plaques and luminal stenosis and can accurately indicates vascular inflammation and cardiovascular risk (17, 18). By drawing spatial changes in perivascular fat attenuation on CCTA, pFAI reflects changes in the size and lipid content of local adipocytes around the coronary artery, directly visualizing and quantifying perivascular inflammation. Inflammation is another critical feature of atherosclerosis (19). Similarly, the “inflammatory theory” of brain disease proposes that the destruction of microcirculation leads to the formation of WMH (16). In addition to the common role of inflammation in the occurrence and development of these two diseases, the brain and the heart both rely on perfusion to meet their high metabolic needs. The regulation of resistance to cerebral microcirculation is a key to maintaining adequate local cerebral blood flow (12). Previous research showed a clear correlation between the CAC volume and poor integrity of white matter microstructure (20). Moreover, plaques with larger volumes in the coronary artery can affect image quality due to patchy and blooming artifacts, leading to errors in the calculation of lumen stenosis, calcification score, and hemodynamic evaluations (21–23). The FFR from invasive coronary angiography (ICA) is the reference standard for detecting disease-specific ischemia, but this test is costly and invasive (24). CT-FFR provides data similar that from invasive FFR, and is used to evaluate the degree of cardiac ischemia using CCTA (25, 26). Therefore, abnormal perfusion in these two organs appear to cause similar pathological changes.

Radiomics can extract quantitative features from images and aid in the diagnosis of multiple disorders, including cardiovascular and cerebrovascular diseases (27, 28). Thus, whole-brain white matter radiomics can be used to predict the progression of WMH (29). Radiomics features obtained from cardiovascular magnetic resonance (CMR) images can detect cardiac changes that are related to chronic cerebral ischemia (30). CCTA can also establish a relationship between the heart and brain. However, the pFAI measurements are only based on the voxel intensity value (31). It is also unclear whether PCAT and myocardial radiomics features can predict WMH progression.

The purpose of the study is to evaluate the value of CCTA-derived markers and radiomics in predicting WMH progression. We compared the predictive value of pFAI with CT-FFR and radiomics signatures and then used the pFAI and CT-FFR, and the radiomics signatures to develop and validate a hybrid model for predicting WMH progression.

## Methods

### Study design and participants

**Figure 1:**
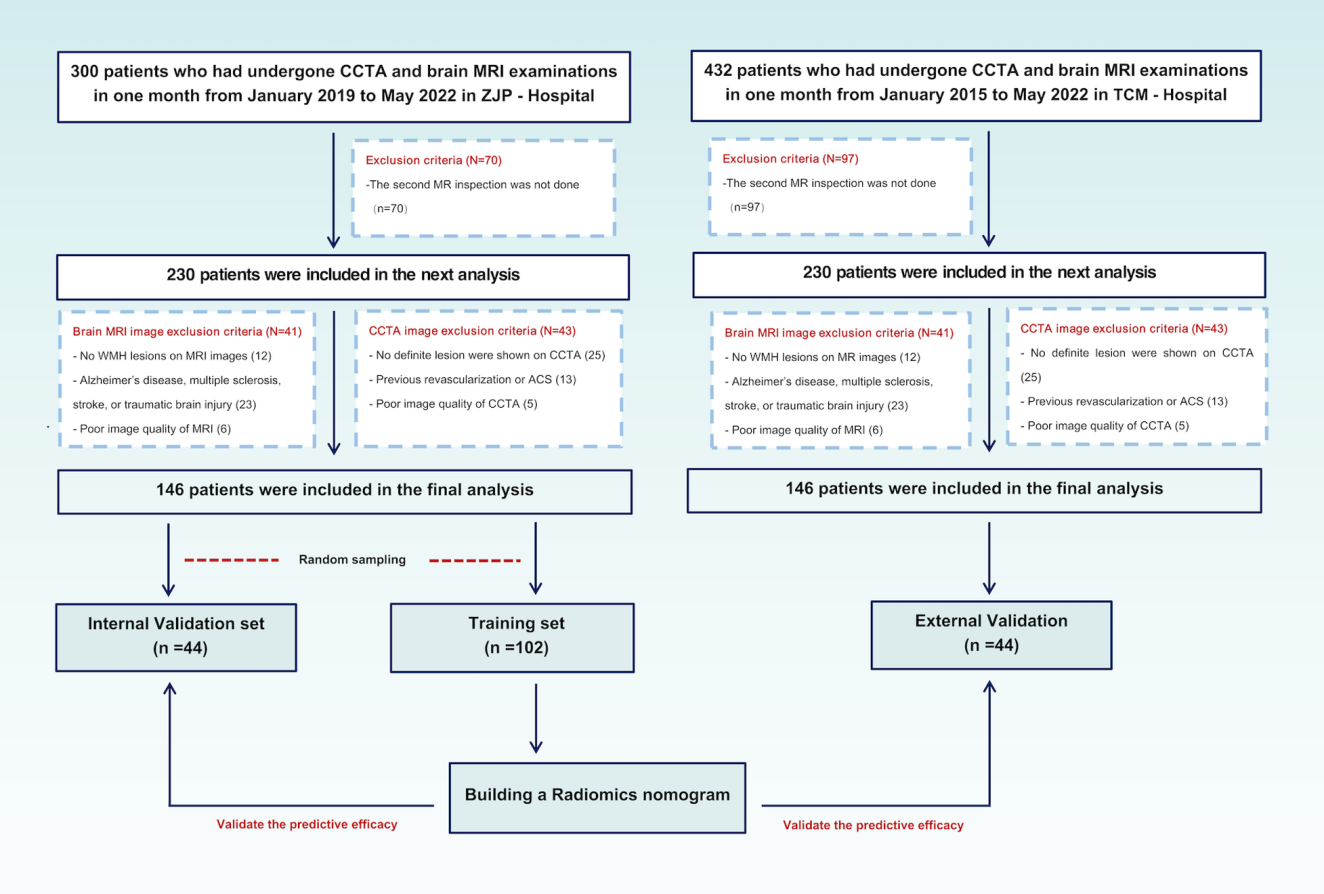
Patients disposition and research design. CCTA, coronary computed tomography angiography; MRI, magnetic resonance imaging; ACS, acute coronary syndrome.

The patients who over 60 years old received CCTA and brain MRI within one month from January 2019 to May 2022 at ZJP Hospital and January 2015 to May 2022 at TCM Hospital were examined. In order to accurately evaluate the WMH progression, patients who underwent second MRI examination after six months were subsequently included. After the application of the inclusion and exclusion criteria (see below), 146 patients from ZJP Hospital were randomly stratified into the training set (*n* = 116, 50 patients with WMH progression), internal validation set (*n* = 30, 13 patients with WMH progression), and 80 patients (51 patients with WMH progression) from TCM Hospital were included into the external validation set. The inclusion criteria were: (1) evidence of WMH based on T2 FAIR and T2-weighted MRI, (2) no lesion attributable to stroke on diffusion-weighted MRI, (3) no signs of Alzheimer's disease, multiple sclerosis, or traumatic brain injury, and (4) no history of myocardial infarction and revascularization. The exclusion criteria were: (1) the presence of a vascular white matter disease, (2) signs of cerebral hemorrhage, (3) no definite lesion signs of calcified or non—calcified plaques and luminal stenosis on CCTA, (4) previous revascularization or ACS, or (5) poor quality of CCTA and MRI images. The inclusion and exclusion process of the patients are shown in Figure 1. This research protocol was approved by the local Ethics Committee of two hospitals.

### Acquisition and analysis of MR images

Brain MRI was both performed using 3.0 T MRI scanner. All brain images were scanned and obtained using an 8-channel head coil 3.0 T MRI scanner (ZJP Hospital: Discovery MR 750, GE Healthcare; TCM Hospital: Siemens Trio 3.0 T) with the same parameter settings. The routine sequences of scanning included T1 weighted, T2 weighted, diffusion weighted imaging and fluid-attenuated inversion recovery (FLAIR). T2 FLAIR and T1 weighted images were used for WMH observation and calculation. The specific parameters and routine sequences of the brain MRI are provided in the Supplementary Material. T2 FLAIR and T1 weighted images were imported into MATLAB (The MathWorks, Inc, Natick, United States) for WMH segmentation and volume calculation. The WMH volume was measured using a 1 mm^3^ spatial dimension of a voxel in each MRI slice. During this process, further automatic segmentation and correction of WMH were carried out, including eliminating non-brain matter and refining WMH segmentation. Images that were considered by both radiologists to have significant segmentation errors were manually segmented and measured again using ITK—SNAP software (http://www.itksnap.org/pmwiki/pmwiki.php) again. The final corrected image was then used for calculation of WMH. The detailed description of the process are shown in Supplementary Figure S1A. Patients were divided into WMH progression and no WMH progression group based on changes of WMH volume. WMH volume progression was defined as an observed increase of more than 0.25 ml in WMH volume, a value derived from the 2015 study of Cho et al. (32). The schematic diagrams of two consecutive brain MRI images of the WMH progression and no-WMH progress groups were shown in Supplementary Figures S1B,C.

### Acquisition and analysis of CCTA images

All CCTA examinations were performed with a CT scanner using 64 detector rows with prospective electrocardiogram (ECG) -gating (ZJP Hospital: Somatom Flash, Siemens Healthineers, Forchheim, Germany; TCM Hospital: Aquilion One, Toshiba Medical, Otawara, Japan) with the same parameter settings. Detailed information about the CCTA are in the Supplementary Material. Patients with high myocardial jeopardy, high grade angina pectoris, or two or three proximal vascular lesions are likely to receive surgical treatment during the follow-up period (33). These surgically treated vessels may affect the acquisition of the CT-FFR and pFAI and the extraction of radiomics features due to metal artifacts and vascular reconstruction. Therefore, we only included three-vessel (left anterior descending artery [LAD], left circumflex artery [LCX], and right coronary artery [RCA]) for patients who did not undergo surgical treatment and had definite lesions. The Gensini scores were used to evaluate the degree of coronary artery stenosis (34). This score was 0 (no stenosis), 1(1%–49% stenosis), 2 (50%–74% stenosis), 3 (75%–99% stenosis), or 4 (100% stenosis). Finally, the total scores of all segments was considered the final score. According to the Gensini scoring system, coronary artery stenosis was finally classified as mild stenosis (1–14 points) or severe stenosis (>14 points). The analysis of CCTA images were independently performed by two experienced radiologists who were unaware of the clinical data.

### Acquisition of CT-FFR and pFAI

**Figure 2:**
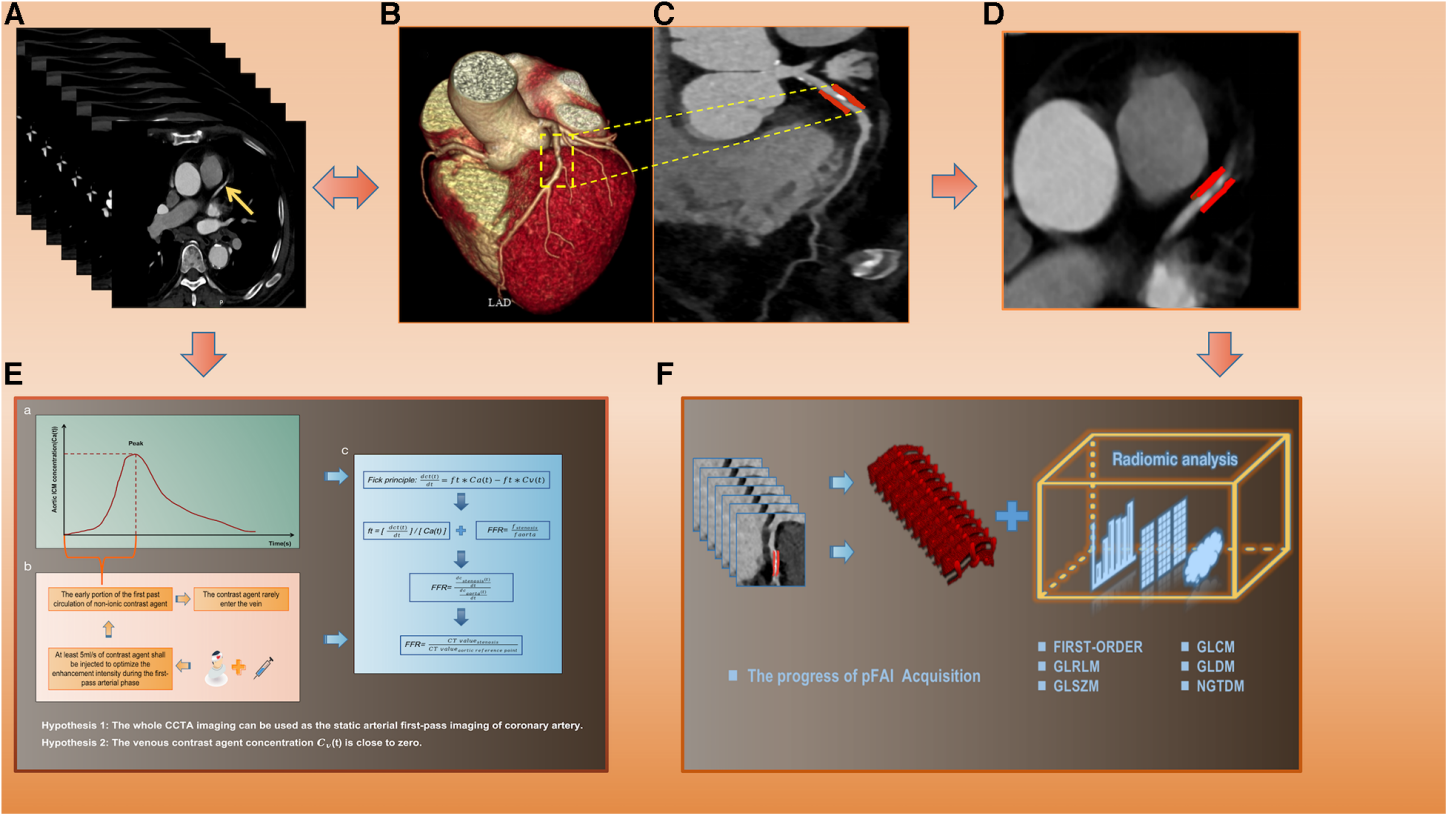
The specific calculation process of CT-FFR and pFAI and radiomics extraction. (**A**) Original CCTA images. (**B,C**) CCTA reconstruction images and lesion segments display. (**D**) PCAT ROI. (**E**) The calculation process and formula of CT-FFR. (**F**) pFAI acquisition and radiomics features analysis. CT-FFR, CT fractional flow reserve; pFAI, percoronary fat attenuation index; CCTA, coronary computed tomography angiography; PCAT, pericoronary adipose tissue.

The two CCTA-derived biomarkers, pFAI and CT-FFR, can effectively reflect CAD from both vascular inflammation and hemodynamics. In our study, we used PHIgo workstations to measure CT-FFR and pFAI based on deep learning methods (version 1.5.1, GE Healthcare). Arterial phase images of CCTA in each patient were imported in DICOM format into the CQK analysis platform of PHIgo (version 1.5.1, GE Healthcare) software for automated segmentation of PCAT and whole myocardium. PCAT around the stenosis lesion on coronary segments (≥2 mm) can be accurately delineated according to the 18-segment guidelines on the arterial phase images. Radiologists A and B evaluated images of all patients for semi-automatic segmentation of PCAT, and manually corrected images with poor segmentation results and recognition errors for stenosis lesions. After the above steps are processed, the CT-FFR and pFAI at the target lesion are calculated using semi-automatic software. The detailed process is shown in the Supplementary Material and Figure 2. Based on threshold values for high risk of CAD (CT-FFR ≤ 0.80 and FAI ≥ −70.1 HU) (35), the minimum CT-FFR and maximum pFAI values of the three-vessel in the follow-up study were included.

### Acquisition and refining of radiomics features

PCAT was defined by voxels with CT attenuation in the range of −190 to −30 HU, and a distance from the outer wall of the coronary artery equal to the diameter of the corresponding horizontal coronary artery (36). When there are multiple vascular lesions, selecting only the largest pFAI among the three vessels for analysis can indicate a higher cardiovascular risk, but there are certain limitations in fully representing the pathological and physiological changes of all vascular lesions. In addition, pFAI is only calculated based on voxel intensity values, and higher-order statistical analysis of spatial relationships from features of radiomics texture can reflect more complex voxel relationships. This is a deeper level of image change that we cannot capture using only pFAI values (37). Therefore, the PCAT around the target segments and whole myocardium were selected as ROIs. If there were multiple lesions in one vessel, the lesion segment with the largest pFAI value was selected. When a patient had multiple vascular lesions, the lesion segment with each vessel's largest FAI value was jointly used as the PCAT-ROI. In order to minimize the central effect of CT images from different hospitals and scanners (38), all CCTA images were pre-processed. A software package that performs quantitative analysis (A.K. software, GE Healthcare) was used to preprocess the CCTA images before extracting the radiomics features. Each sequence of the images is resampled to a resolution of 1 × 1 × 1 mm^3^ through linear interpolation and the gray level of the images needs to be discretized and normalized to 32 orders. The preprocessing of images, automatic segmentation of ROIs and radiomics feature extraction were shown in the Supplementary Material. We first evaluated the the repeatability of features in the intra- and inter-observers during the feature extraction process using intraclass correlation coefficients(ICCs), retaining features with high reproducibility with ICCs greater than 0.75 (39). Then, we used ComBat to normalize and gather the data distributions to eliminate the central effects of two centers (Supplementary Figure S2). Next, Mann-Whitney U analysis and Elastic Net Regression were used to filter out redundant signatures. The specific formula for Elastic Net Regression and optimal signatures was shown in the Supplementary Material.

### Model construction and validation

**Figure 3:**
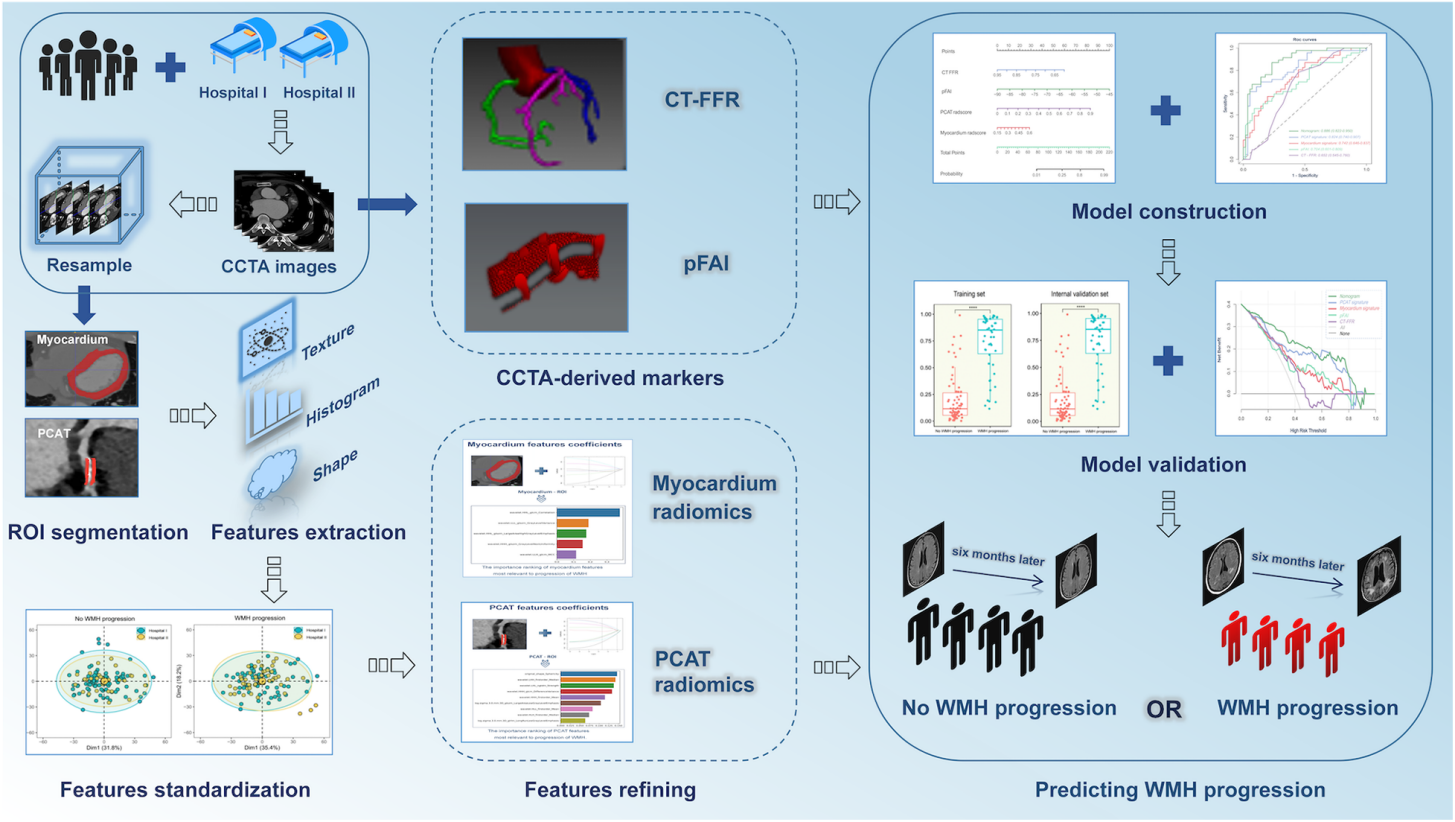
Procedures used for radiomics analysis. CCTA, coronary computed tomography angiography; CT-FFR, CT fractional flow reserve; pFAI, pericoronary fat attenuation index; WMH, white matter hyperintensity.

Univariate logistic regression analysis was used to select significant factors from potential predictors (age, gender, BMI, hypertension, diabetes mellitus, hyperlipidemia, smoking, alcohol intake, the number and grade of vascular stenosis, pFAI, CT-FFR, PCAT and myocardium radiomics signatures), and then the screened factors were included in the multivariate analysis to finally determine the independent predictors of WMH progression for model construction. We first used different machine learning algorithms [LR (Logistic Regression), SVM (Support Vector Machine), Random Forest (RF), k-nearest neighbor (KNN) and eXtreme Gradient Gradient Boosting Machine (XGBoost)] to construct radiomics models. LR was a mature and powerful supervised classification method. It could be considered as an extension of ordinary regression and could only model the outcome events of binary variables. However, it could help discover the possibility that new instances belong to a certain class (40). SVM algorithm could classify linear and non-linear data. It first maped each data item to an n-dimensional feature space, where *n* represented the number of features. Then, SVM recognized hyperplanes that divided the data items into two categories, while maximizing the edge distance between the two categories and minimizing classification errors (41). Random Forest (RF) was an ensemble classifier composed of many DTs, and deep growing DTs often led to overfitting of training data, resulting in small changes in input data and high changes in classification results. When using this algorithm, different parts of the training dataset needed to be used to train different DTs of RF. Since the RF algorithm considered the results of many different DTs, it could reduce the variance generated by considering a single DT for the same dataset (42). KNN algorithm was one of the earliest and very simple classification algorithms. The KNN algorithm did not need to consider probability values. KNN was particularly suitable for multi classification problems and was relatively easy to understand and implement (43). XGBoost was a supervised algorithm that belongs to ensemble learning algorithms. It was a scalable and convenient Gradient Boosting algorithm that could build models in parallel. The XGBoost algorithm had certain advantages in preventing model overfitting. Each algorithm had unique advantages, and we compared multiple algorithms to find the optimal algorithm for constructing unitary and hybrid models based on single and all independent risk factors of WMH progression. The area under the curve (AUC) from receiver operator characteristic curve (ROC) analysis was used to evaluate the accuracy of different models. Finally, the goodness-of-fit of the hybrid model was evaluated by using calibration curve and nonparametric test, and decision curve analysis (DCA) was used to evaluate the clinical value of different models. Figure 3 shows the workflow used for the radiomics analysis.

### Statistical analysis

Statistical analyses were performed using R software (version 3.5.0), SPSS software(version 17.0, Armonk, NY) and Python (version 3.5). Continuous variables were expressed as means ± standard deviations. Categorical variables were compared using the chi-square test. The normality of distribution was assessed using the Kolmogorov-Smirnov test, and variables were then compared using the *t*-test (normal distributions) or the Mann-Whitney test (non-normal distributions). The intra- and inter-observer reproducibility of extracted features was evaluated using the ICCs. For features extracted from each ROI, the Mann-Whitney *U* test and Elastic Net Regression were used for filtering redundant and irrelevant signatures. The predictions of different models were evaluated according to AUC, accuracy, and specificity. Compare the ROC curves of different models by using the nonparametric method of DeLong test (44). A *p*-value below 0.05 was considered significant.

## Results

### Patient characteristics and study design

We finally enrolled 146 and 80 patients who had coronary artery stenosis and WMH in the two hospitals. We used the second MRI results to evaluate changes of WMH volume. The WMH volume progressed in 63 patients in ZJP Hospital and 51 patients in TCM Hospital (Supplementary Table S1). Gender, age, BMI, hypertension, diabetes mellitus, hypertension, tobacco smoking, alcohol use, and the number and degree of stenotic vessels were not significantly different between the “WMH progression” group and the “no WMH progression” group for the training set, internal validation set and external validation set. However, the CCTA-derived markers and the radiomics scores were significantly different for the “WMH progression” group and the “no WMH progression” group in the training set and external validation set (both *p* < 0.05; Table 1).

**Table 1:** Clinical characteristics and radiomics scores of the “WMH progression” group and the “no WMH progression group” in the training set, internal validation set, and external validation set.

Characteristics	Training set		*P*	Internal validation set		*P*	External validation set		*P*
	WMH Progression	No WMH Progression		WMH Progression	No WMH Progression		WMH Progression	No WMH Progression	
	(*n* = 50)	(*n* = 66)		(*n* = 13)	(*n* = 17)		(*n* = 51)	(*n* = 29)	
Demographics									
Age [y]^a^	69.7 ± 5.3	69.9 ± 6.4	0.931	77.9 ± 6.2	73.1 ± 6.7	0.051	75.3 ± 10.2	70.9 ± 13.0	0.094
Male sex [*n*]^b^	35 (70.0%)	44 (66.7%)	0.703	10 (76.9%)	9 (52.9%)	0.177	28 (54.9%)	14 (48.3%)	0.568
Cardiovascular risk factors									
BMI [kg/m^2^]^a^	22.6 ± 4.3	23.7 ± 3.4	0.129	24.4 ± 2.6	24.2 ± 3.1	0.876	22.9 ± 3.73	23.9 ± 3.1	0.214
Hypertension [*n*]^b^	35 (70.0%)	38 (57.6%)	0.170	10 (76.9%)	12 (70.6%)	0.697	32 (62.7%)	20 (69.0%)	0.575
Diabetes mellitus [*n*]^b^	17 (34.0%)	18 (27.3%)	0.434	7 (53.8%)	7 (41.2%)	0.491	19 (37.3%)	13 (44.8%)	0.506
Hyperlipidemia [*n*]^b^	3 (6.0%)	7 (10.6%)	0.381	1 (7.7%)	2 (11.8%)	0.713	9 (17.6%)	12 (41.4%)	**0**.**020**
Smoking in past 5 years [*n*]^b^	23 (46.0%)	25 (37.9%)	0.379	5 (38.5%)	2 (11.8%)	0.087	9 (17.6%)	5 (17.2%)	0.963
Alcohol intake in past 5 years [*n*]^b^	15 (30.0%)	19 (28.8%)	0.887	5 (38.5%)	2 (11.8%)	0.087	8 (15.7%)	4 (13.8%)	0.820
Month [*n*]^a^	15 ± 5	14 ± 4	0.228	13 ± 4	15 ± 4	0.218	14 ± 5	13 ± 5	0.658
CCTA parameters									
Stenotic vessels number [*n*]^b^			0.331			0.254			0.114
1	16 (32.0%)	30 (45.5%)		2 (15.4%)	7 (41.2%)		16 (31.4%)	11 (37.9%)	
2	16 (32.0%)	18 (27.3%)		4 (30.8%)	5 (29.4%)		20 (39.2%)	5 (17.2%)	
3	18 (36.0%)	18 (27.3%)		7 (53.8%)	5 (29.4%)		15 (29.4%)	13 (44.8%)	
Stenosis classification^b^			0.702			0.632			0.559
1	24 (48.0%)	36 (54.5%)		6 (46.2%)	8 (47.1%)		17 (33.3%)	13 (44.8%)	
2	18 (36.0%)	19 (28.8%)		4 (30.8%)	5 (29.4%)		23 (45.1%)	10 (34.5%)	
3	8 (16.0%)	11 (16.7%)		3 (23.1%)	4 (23.5%)		11 (21.6%)	6 (20.7%)	
pFAI [HU]^a^	−63.5 ± 5.4	−69.3 ± 5.7	**<0.001**	−60.1 ± 7.2	−68.3 ± 6.2	**0**.**002**	−71.3 ± 7.1	−79.3 ± 8.0	**<0.001**
CT-FFR^a^	0.7 ± 0.1	0.8 ± 0.1	**0**.**033**	0.8 ± 0.0	0.8 ± 0.1	0.077	0.7 ± 0.1	0.8 ± 0.1	**0**.**005**
Myocardium-rad score^a^	0.4 ± 0.1	0.3 ± 0.1	**<0.001**	0.4 ± 0.1	0.3 ± 0.1	**0**.**022**	0.4 ± 0.1	0.3 ± 0.1	**0**.**002**
PCAT-rad score^a^	0.5 ± 0.2	0.2 ± 0.2	**<0.001**	0.5 ± 0.3	0.3 ± 0.2	**0**.**015**	0.4 ± 0.2	0.3 ± 0.2	**0**.**001**

BMI, body mass index; pFAI, pericoronary fat attenuation index; CT-FFR, CT fractional flow reserve; WMH, white matter hyperintensity; rad score, radiomics score.

^a^ Presented as mean ± standard deviation, and Student's *t* test was performed to compare these variables.

^b^ Presented as frequencies and percentages, and Chi-square test was used for the comparisons of these variables.

Bold values represent *P* < 0.05, with significant statistical differences.

### Development of radiomics signatures and assessment of performance

**Figure 4:**
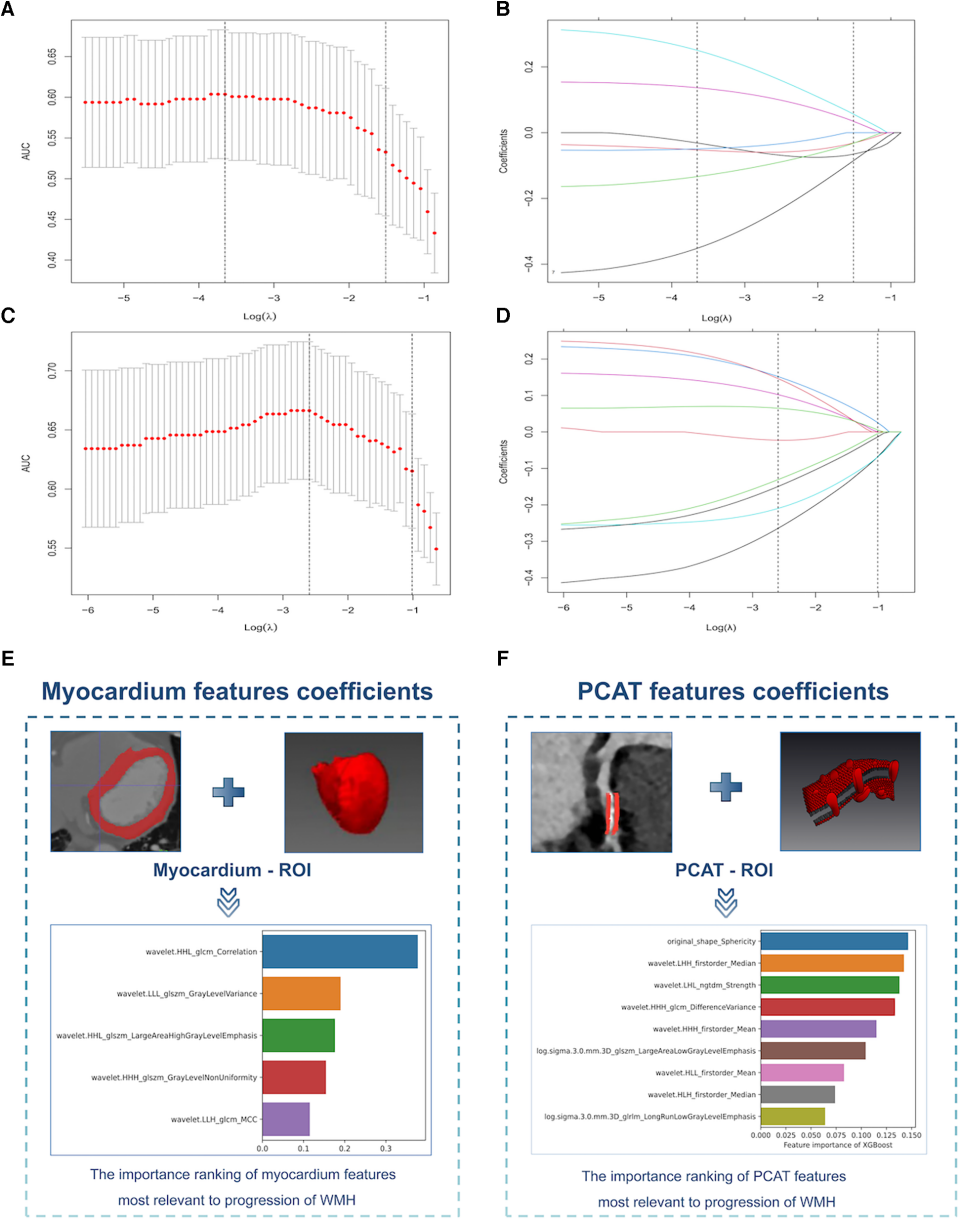
Selection of myocardium and PCAT radiomics signatures for the prediction models. (**A,C**) Myocardium (top) and PCAT (bottom) signatures selection by Elastic Net Regression with the optimal penalization coefficient lambda (*λ*) using 10-fold cross-validation and the minimal criteria process. The x-axis shows Lambda (*λ*), and the y-axis shows the model AUC. (**B,D**) Elastic Net Regression coefficient profiles of the myocardium signatures (top) and PCAT signatures (bottom). (**E,F**) Importance ranking of the myocardium signatures (left) and PCAT signatures (right) that had the greatest correlations with WMH progression. PCAT, pericoronary adipose tissue, WMH, white matter hyperintensity.

**Figure 5:**
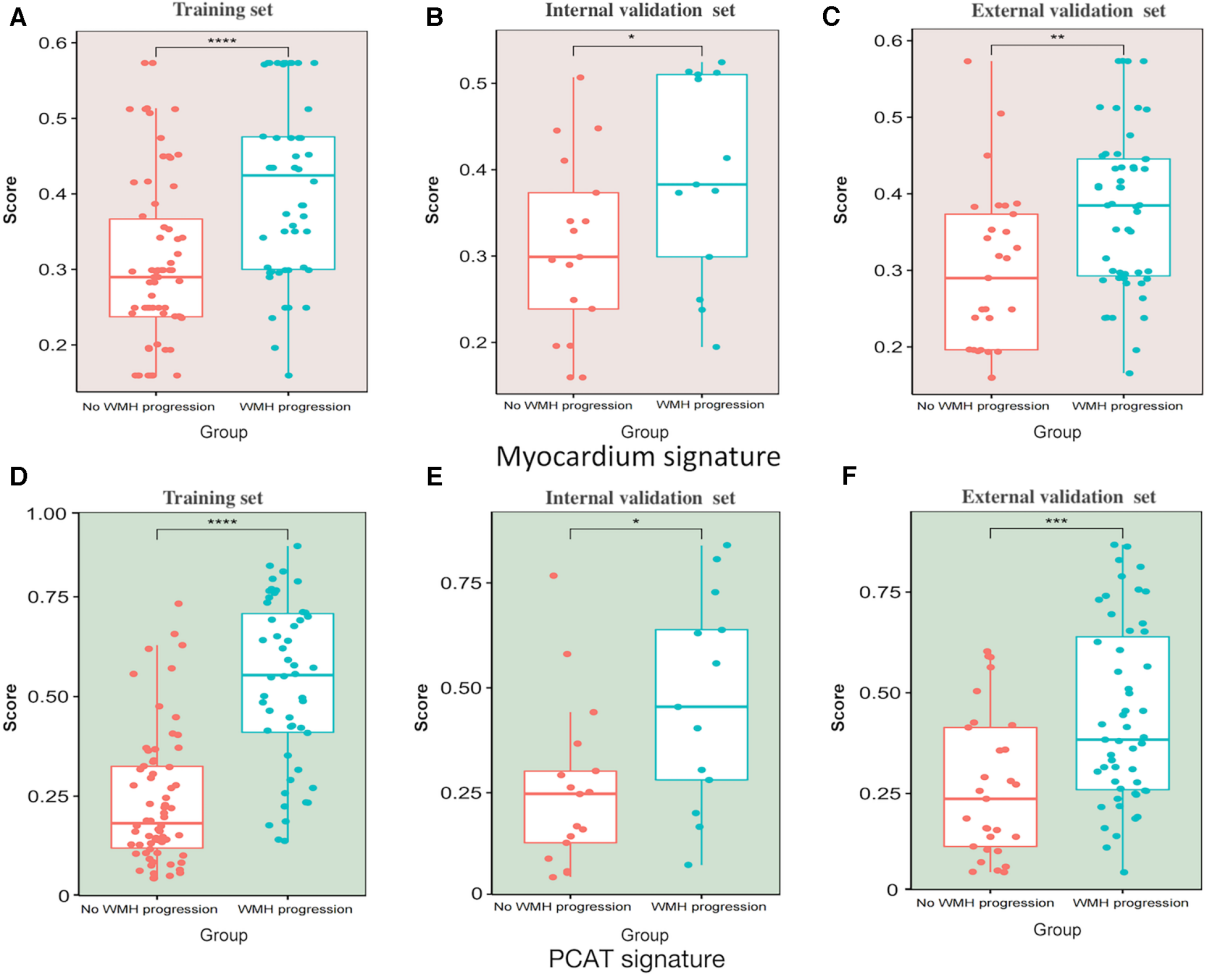
Scatter box plot of radiomics signatures scores. (**A–C**) Myocardium signatures radiomics scores in the “WMH progression” group and the “no WMH progression” group in the training set, internal validation set and external validation set. (**D–F**) PCAT signatures radiomics scores in the “WMH progression” group and the “no WMH progression” group in training set, internal validation set and external validation set. The horizontal line in the box represents the median of the signatures score, the upper and lower borders of the box represent the upper and lower quartiles of signatures score, and the scattered points around the box represent the distribution position of each radiomics signatures score. PCAT, pericoronary adipose tissue; WMH, white matter hyperintensity. *Represents *p* < 0.05, **represents *p* < 0.01, *** represents *p* < 0.001, and ***represents *p* < 0.0001.

We extracted 1,073 features from the PCAT-ROI and myocardium-ROI by PyRadiomics. After standardisation of scanner type and location and using specific feature reduction methods, we selected 5 myocardial and 9 PCAT radiomics signatures that were statistically different between those with and without WMH progression (Figure 4). The results indicated there were statistically significant differences in the radiomics scores for the “WMH progression” group and the “no WMH progression” group in the training, internal validation and external validation set (*p *< 0.05) (Figure 5). By comparing the AUC of myocardium and PCAT radiomics signatures constructed by different algorithms of LR, SVM, RF, KNN and XGBoost, it was demonstrated that PCAT radiomics signatures and XGBoost algorithm have better prediction efficiency in the training, internal validation and external validation set (AUC: 0.862 [95% CI: 0.788–0.924], 0.760 [95% CI: 0.570–0.923] and 0.731 [95% CI: 0.600–0.836]) in Supplementary Table S2 and Supplementary Figure S3.

### Development of a hybrid model

The results demonstrated that CT-FFR, pFAI, PCAT and myocardium radiomics scores were independent predictors of WMH progression (all *p* < 0.05, Table 2).

**Table 2:** Independent predictors of WMH progression based on logistic regression analysis.

	Univariate logistic regression		Multivariate logistic regression	
	OR (95% CI)	*P*	OR (95% CI)	*P*
Age	1.00 (0.94–1.07)	0.930	NA	NA
Gender	0.86 (0.39–1.89)	0.703	NA	NA
BMI	0.93 (0.84–1.02)	0.176	NA	NA
Hypertension	1.72 (0.79–3.74)	0.172	NA	NA
Diabetes mellitus	1.37 (0.62–3.05)	0.435	NA	NA
Hyperlipidemia	0.54 (0.13–2.19)	0.387	NA	NA
Smoking	1.40 (0.66–2.95)	0.380	NA	NA
Alcohol	1.06 (0.47–2.37)	0.887	NA	NA
Stenotic vessels number	1.38 (0.83–2.14)	0.159	NA	NA
Stenosis classification	0.92 (0.32–2.61)	0.871	NA	NA
pFAI	1.23 (1.12–1.34)	**<0** **.** **001**	1.16 (1.04 −1.29)	**0.006**
CT-FFR	0.04 (0.00–0.63)	**0**.**016**	0.000002 (1.1497E-10–0.049)	**0.011**
Myocardium—rad score	2.10 (1.48–2.97)	**<0.001**	1.62 (1.01–2.60)	**0.044**
PCAT—rad score	2.04 (1.59–2.61)	**<0.001**	1.921 (1.434–2.575)	**<0.001**

WMH, white matter hyperintensity; BMI, body mass index; pFAI, pericoronary fat attenuation index; CT-FFR, CT fractional flow reserve; PCAT, pericoronary adipose tissue; rad score, radiomics score; OR, odds ratio.

Bold values represent *P* < 0.05, with significant statistical differences.

**Figure 6:**
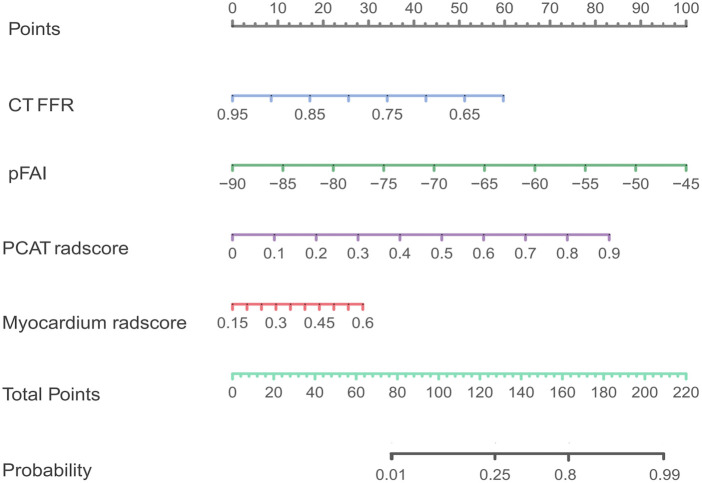
The variables included in the hybrid model are presented as a nomogram.

**Figure 7:**
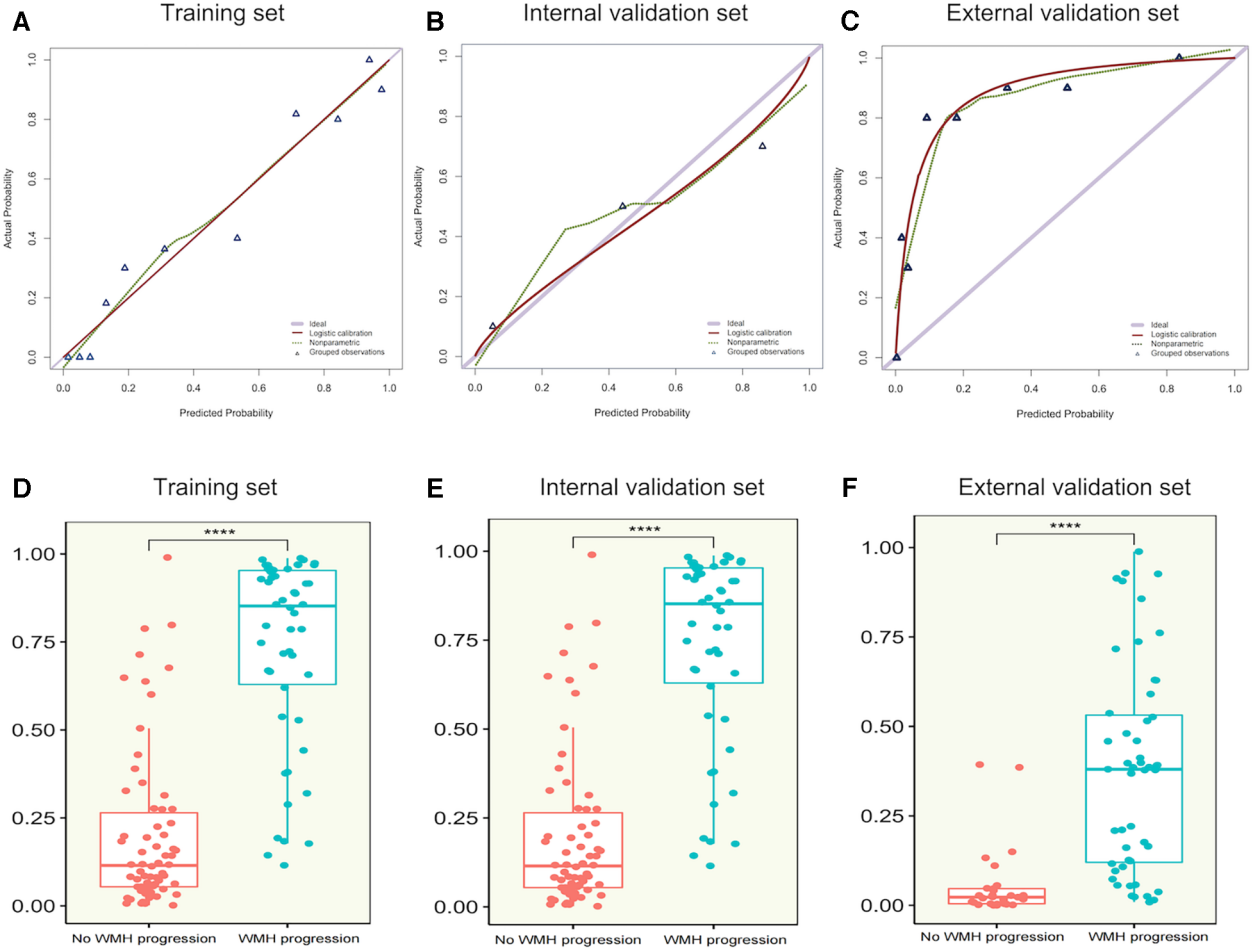
(**A–C**) calibration curves of hybrid model for WMH progression in the training set, internal validation set and external validation set. The x-axis represents the predicted probability of WMH progression estimated by the nomogram, and the y-axis represents the actual WMH progress probability. The red solid line represents logistic regression calibration, and the green dashed line represents nonparametric testing. The closer the red solid fit to the green dashed, the higher the accuracy of nomogram for predicting WMH progression. The triangle represents the grouped observations. (**D–F**) The predictive probability of the hybrid model in the “WMH progression” group and the “no WMH progression” group in the training set, internal validation set and external validation set. The horizontal line in the box represents the median of the predictive probability, the upper and lower borders of the box represent the upper and lower quartiles of predictive probability, and the scattered points around the box represent the distribution position of predictive probability. CT-FFR, CT fractional flow reserve; pFAI, pericoronary fat attenuation index; PCAT, pericoronary adipose tissue; radscore, radiomics score. *Represents *p* < 0.05, ** represents *p* < 0.01, ***represents *p* < 0.001, and ***represents *p* < 0.0001.

After comparing different machine learning algorithms, we constructed a hybrid model using the XGBoost algorithm based on CT-FFR, pFAI, PCAT and myocardium radiomics scores. The weight scores of each independent predictors in the hybrid model were shown in a nomogram (Figure 6). To verify the accuracy of hybrid model, we plotted calibration curves and assessed the goodness of fit of the hybrid model. The results showed that the predictions of hybrid model were highly consistent with the observed values. The nonparametric test showed that prediction efficiency was not statistically different for the training set, internal validation set and external validation set (Figures 7A–C; *p* < 0.05). We observed significant differences between the “WMH progression” group and the “no WMH progression” group by assessing the predictive ability of hybrid model in the training, internal validation and external validation set (Figures 7D–F).

### Development and validation of different models

**Figure 8:**
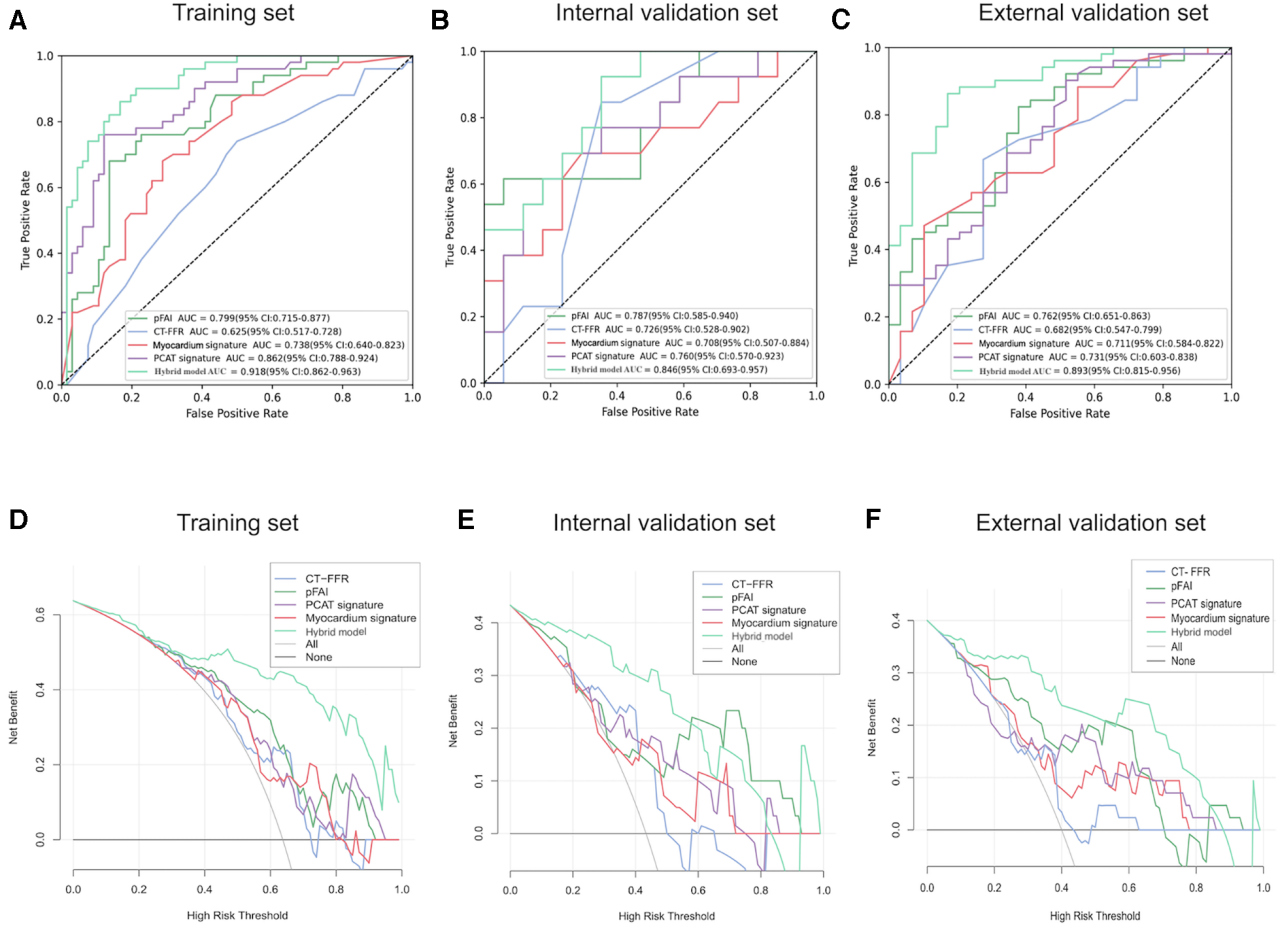
Diagnostic performance of different models in predicting WMH progression. (**A–C**) ROC curves for different models for prediction of WMH progression in the training set, internal validation set and external validation set. (**D–F**) DCA of different models from the training set, internal validation set and external validation set. The horizontal black line indicates the net benefit assuming that no patients have WMH progression and the smooth gray line indicates the net benefit assuming that all patients have WMH progression. PCAT, pericoronary adipose tissue; pFAI, pericoronary fat attenuation index; CT-FFR, CT fractional flow reserve; ROC, receiver operating characteristic; DCA, decision curve analysis.

We also built unitary models based on the CCTA-derived markers and radiomics signatures in sequence. Analysis of the external validation set demonstrated that the pFAI had greater performance than the CT-FFR for predicting WMH progression (AUC: 0.762 [95% CI: 0.651–0.863] vs. 0.682 [95% CI: 0.547–0.799]; *p* < 0.05). Comparing the two radiomics signatures showed that the PCAT signatures had a slightly higher AUC value than myocardial signatures [AUC: 0.731 (95% CI: 0.603–0.838) vs. 0.711, 95% CI: 0.584–0.822]; *p* > 0.05) (Figures 8A–C). Then, we found that the hybrid model with all parameters can show the optimal prediction performance in the training set, internal validation set and external validation set (AUC: 0.918 [95% CI: 0.862–0.963, 0.846 [95% CI: 0.693–0.957 and 0.893 [95% CI: 0.815–0.956) (Figures 8A–C). Based on the DeLong test, we found significant differences between the hybrid model and unitary models (*p* < 0.001). The hybrid model also had high accuracy, sensitivity and specificity in the training, internal validation and external validation set (Table 3). Furthermore, DCA showed that the hybrid model provided more net benefit than the “all treatment” or “no treatment” options in the training, internal validation and external validation set (Figures 8D–F). When the high risk threshold was less than 0.82 (internal validation set) and 0.84 (external validation set), the hybrid model had a greater net benefit than other models.

**Table 3:** The diagnostic performance of different models.

	Model	AUC (95% CI)	Accuracy	Sensitivity	Specificity
Training	pFAI	0.80 (0.75–0.85)	0.78	0.68	0.86
	CT-FFR	0.63 (0.59–0.67)	0.60	0.74	0.50
	Mycardium signature	0.74 (0.70–0.78)	0.70	0.68	0.71
	PCAT signature	0.86 (0.81–0.89)	0.83	0.76	0.88
	Nomogram	0.92 (0.90–0.94)	0.85	0.86	0.83
Internal validation	pFAI	0.79 (0.49–0.88)	0.63	0.62	0.65
	CT-FFR	0.73 (0.71–0.82)	0.73	0.85	0.65
	Mycardium signature	0.71 (0.56–0.81)	0.70	0.69	0.71
	PCAT signature	0.76 (0.67–0.84)	0.70	0.54	0.82
	Nomogram	0.85 (0.70–0.94)	0.73	0.85	0.65
External validation	pFAI	0.76 (0.65–0.86)	0.54	0.29	0.97
	CT-FFR	0.68 (0.55–0.80)	0.58	0.49	0.72
	Mycardium signature	0.71 (0.58–0.82)	0.64	0.63	0.66
	PCAT signature	0.73 (0.60–0.84)	0.56	0.47	0.72
	Nomogram	0.89 (0.82–0.96)	0.69	0.55	0.93

pFAI, pericoronary fat attenuation index; CT-FFR, CT fractional flow reserve; PCAT, pericoronary adipose tissue.

## Discussion

We compared the predictive performance of PCAT and mycocardium radiomics signatures by using different machine learning algorithms, and constructed a hybrid model for comprehensive comparison with pFAI, CT-FFR and radiomics signatures. The results indicate that CCTA-derived markers and radiomics signatures can effectively predict the WMH progression. The occurrence and progress of WMH can lead to a series of subsequent degenerative diseases such as fiber demyelination and cortical damage (45). These changes can cause irreversible damage to the microstructure of the brain and, to a certain extent, have certain indicators of poor prognosis for patients (46). An early analysis based on the UK Biobank suggested that lower left ventricular (LV) ejection fraction was associated with brain structural abnormalities such as lower gray matter volume and greater WMH volume (47). In fact, many vascular risk factors, including smoking, hypertension, diabetes, obesity and lack of exercise, have been proved to be related to poor performance in brain (48). Among cardiovascular risk factors, hypertension has a stronger correlation with WMH and may damage brain microcirculation (49). Another report from the Framingham cohort study also suggested that hypertension and smoking were associated with higher WMH burden (50). In a large Chinese cohort of 4,683 subjects, it was found that high low density lipoprotein (LDL) cholesterol was associated with high WMH (51). Therefore, it is essential to implement appropriate interventions that control vascular risk factors to prevent WMH progression, such as timely control of hypertension and dyslipidemia (16, 52). In CAD, pFAI is a marker of coronary arteritis and CT-FFR is an imaging marker of defective hemodynamics. The co-occurrence of inflammation and diminished blood perfusion has unique pathophysiological effects in the development of cerebrovascular diseases (53–55). Research has shown that poorer LV function and decreased arterial compliance were closely related to adverse brain characteristics, which can provide a research basis for the theory of vascular hypoperfusion, which is closely related to cerebral hypoperfusion and microvascular plaque accumulation (15). Our study showed that the pFAI had greater predictive value than the CT-FFR, suggesting a hidden inflammatory pathway between the cardio-cerebral diseases. This result is consistent with the interpretation of Moroni et al. (12), who concluded these two microcirculatory diseases were pathogenically connected. In fact, some common pathophysiological features of the brain and heart, such as inflammation theory and blood flow perfusion, may be mainly related to the vascular function of the two organ systems. There is indeed a certain comorbidity mechanism between the two diseases in terms of inflammation and blood flow perfusion.

Vascular lesions may cause multiple myocardial changes such as myocardial ischemia, decreased myocardial flow, and changes in myocardial contractile function, followed by indirect changes in some pathogenic pathways between the heart and brain (12, 56, 57). The pFAI can reflect perivascular inflammation and cardiovascular disease (18). However, the pFAI is only based on voxel intensity and does not consider the complex relationships among voxels (31). The key difference between machine learning algorithms and traditional methods was that machine learning algorithms can learn from observations, enabling perform the mapping from features to labels at the image level and create a model that can summarize information into a new, previously unseen inputs (58). Therefore, we used radiomics to extract quantitative information and then selected radiomics signatures that were related to the clinical results to build prediction models by different algorithms (59). After comparing the two kinds of radiomics signatures, the PCAT signatures were better than the myocardium signatures in predicting WMH progression, and the Xgboost algorithm showed superior predictive performance. When comparing CCTA-derived markers and radiomics signatures, we found that the PCAT signatures and pFAI both have similar performance in predicting WMH progression, both higher than myocardium signatures and CT-FFR.

Shu et al. previously predicted the progression of any WMH, periventricular WMH, and deep WMH using radiomics signatures (AUC: 0.714 [any WMH], 0.697 [periventricular WHH], and 0.717 [deep WMH]. By comparing the predictive effects of different signatures on WHH progression, we found that the predictive efficiency of radiomics features extracted from the entire white matter of the brain was higher than that extracted from the entire myocardium [AUC: 0.0.717 (95% CI: 0.603–0.838) vs. 0.711, 95% CI: 0.584–0.822]), but when compared with the PCAT signatures, the PCAT signatures showed a higher predictive ability [AUC: 0.731 (95% CI: 0.603–0.838)] for predicting WMH progression. Our study is the first to show that PCAT can be used to predict WMH progression. The another major innovation is the demonstration that a hybrid model constructed by CCTA was a tool for identifying WMH progression. In addition, we found that the pFAI was a better predictor of WMH progression than the CT-FFR, and the results suggested that heart-related inflammatory changes are more sensitive indicators of changes in WMH than hemodynamic markers. Another study that examined predictors of myocardial ischemia also found a difference between these two markers (60).

There are also many indicators that play an important role in reflecting perivascular inflammation and cardiovascular disease. Hypersensitive C-reactive protein (hs-CRP), which is currently a representative biomarker for detecting inflammation in CAD (61). hs-CRP mediated chronic inflammation is an independent predictor of coronary microvascular dysfunction in patients with ischemic heart disease (62). In addition, vulnerable plaques, as a characteristic inflammatory process of damage and anti-damage, are also closely related to vascular inflammation. Furthermore, there are also many factors related to cardiovascular disease. For example, indicators such as calcified plaque score, degree of luminal stenosis, and left ventricular ejection fraction (LVEF) all have a suggestive effect on cardiovascular risk factors (63, 64). There are also some basic physical indicators that are cardiovascular related risk factors. For example, obesity induces the aggregation of macrophages into perivascular adipose tissue, inhibits the release of vasodilators such as hydrogen sulfide from endothelial cells and smooth muscle cells, and can affect coronary microvascular dilation function, leading to cardiovascular disease (65). On the other hand, aging is also one of the important risk factors for cardiovascular diseases (66). Therefore, it is not enough for us to focus solely on exploring WMH progression based on two imaging markers obtained from CCTA, and further joint evaluation of multiple related factors is needed. In addition, as the impact of cerebral blood flow perfusion on WMH is crucial, we should further explore the correlation between cerebral blood flow perfusion and WMH.

Our study also had certain limitations. First, the population included in our study was relatively small. However, there was no significant difference in sample size between the WMH progression and no-WMH progression group in our study population, so the results had reference significance. Secondly, our determination of WMH progression was based on measurements at two time points. Ideally, this assessment should be based on many measurements over time. In addition, the time interval between two brain MRI examinations was determined to be no less than 6 months, which may have a certain bias in the evaluation of WMH progression due to the short interval time. Regardless, the hybrid model captured the presence of a pathogenic pathway. Thirdly, there were inevitably some biases in the semi-automatic segmentation and volume calculation of WMH using MATLAB software. However, all results were evaluated and corrected by physicians, and the method of obtaining WMH volume using MATLAB has been used multiple times in some other WMH related studies (29, 67). Therefore, the calculation of WMH volume in our study has some reliability. Finally, we used deep learning software and machine learning methods to build models. So it still needed some time to be promoted and applied in clinical practice.

## Conclusions

The CCTA-derived markers and radiomics signatures were reliable indicators for predicting WMH progression. We found that the pFAI was superior to the CT-FFR, and the PCAT signatures was superior to the mycocardium signatures for predicting WMH progression. A hybrid model combining pFAI, CT-FFR, and radiomics features is a potential use for identifying patients with WMH progression in elder coronary heart disease populaiton.

## Data Availability

The original contributions presented in the study are included in the article/**Supplementary Material**, further inquiries can be directed to the corresponding author.
