# Supplementary material for: Hybrid model of CT-fractional flow reserve, pericoronary fat attenuation index and radiomics for predicting the progression of WMH: a dual-center pilot study
Source: Front Cardiovasc Med. 2023 Dec 19;10:1282768. doi: 10.3389/fcvm.2023.1282768 (PMC10766365; doi:10.3389/fcvm.2023.1282768)
Supplement: Supplementary file 1 [file Datasheet1.pdf]

Supplemental information

Hybrid model of CT-fractional flow reserve, pericoronary fat attenuation index and radiomics for predicting the progression of WMH: a Dual-Center pilot study

1. Specific parameters of brain MRI scanning

All brain images were scanned and obtained using an 8-channel head coil 3.0T MRI scanner (ZJP Hospital : Discovery MR 750, GE Healthcare; TCM Hospital: Siemens Trio 3.0T) with the same parameter settings. The routine sequences of scanning included T1 weighted, T2 weighted, diffusion weighted imaging and fluid-attenuated inversion recovery (FLAIR). T2 FLAIR and T1 weighted images were used for WMH observation and calculation, with specific parameters as follows: repetition time (TR) of 9000 ms, echo time (TE) of 120 msec, field of view (FOV) of 220 × 220 cm, matrix of 256 × 256, flip angle of 160°, echo chain of 18, bandwidth of 50, thickness of 5 mm, and interslice gap of 1.5 mm. T1 FLAIR with specific parameters as follows: TR of 1750 ms, TE of 24 msec, FOV of 220 × 220 cm, matrix of 256 × 256, flip angle of 111°, echo chain of 10, bandwidth of 31.25, thickness of 5 mm, and interslice gap of 1.5 mm. DWI were used for small lumens observation, with specific parameters as follows: TR of 3071 ms, the minimum TE, FOV of 220 × 220 cm, matrix of 160 × 192, thickness of 5 mm, interslice gap of 1.5 mm, and b value of 0, 1000. The sequences and parameters of the second MR scan of patients at the follow-up were the same as the above sequences.

2. Example diagram of automatic software segmentation WMH process

In the process of segmentation of WMH, SPM12 (https://www.fil.ion.ucl.ac.uk/spm/software/spm12/) was first run in MATLAB (The MathWorks, Inc, Natick, United States) to register T2-FLAIR and T1 weighted images. According to the lesion prediction algorithm (LPA) in the LST (www.statistical-modelling.de/lst.html) toolbox of SPM12, WMH was automatically segmented using the lesion prediction algorithm for 2D images. The WMH volume was measured using a 1 mm^3 spatial dimension of a voxel in each MRI slice. In this process, further automatic segmentation and correction of WMH were carried out, including eliminating nonbrain matter and refining WMH segmentation. LPA does not require users to set special parameters, and the segmentation effect is faster and more sensitive than the lesion growth algorithm (LGA). In order to minimize the excessive segmentation of LPA, two experienced radiologists (8 years and 5 years of radiology neurologists respectively) independently observed the results. Images that were considered by both radiologists to have significant segmentation errors were manually segmented and measured using itk - snap software (http://www.itksnap.org/pmwiki/pmwiki.php) again. The final corrected image is used for WMH calculation. To display the temporal change of WMH, the volume of WMH on FLAIR images was measured at baseline and at follow-up.

3. Details of scan parameters and CCTA

All CCTA examinations were performed with a CT scanner using 64 detector rows with prospective electrocardiogram (ECG) -gating (ZJP Hospital: Somatom Flash, Siemens Healthineers, Forchheim, Germany; TCM Hospital: Aquilion One, Toshiba

Medical, Otawara, Japan) with the same parameter settings. The tube voltage of the CCTA was a fixed value of 100kV, and the tube current was adjusted based on the patient's BMI, with a reference range of 350-500 mA. During scanning, the scavenging range was set to 1 cm below the tracheal bulge to the level of the cardiac diaphragm. The frame rate is set to 0.35 s/circle, the reconstruction layer thickness is 0.5 mm, and the reconstruction layer interval is 0.25 mm. The image acquisition of CCTA was performed by ECG gated scanning technology, HR < 65 hpm, single sector recombination was implemented, the time of exposure was 70%-80% R-R interval, one cardiac cycle was collected; HR ≥ 65 bpm, multi-sector recombination was implemented, the time of exposure was 30%-80% R-R interval, 65 < HR < 80 BPM was used to collect two cardiac cycles, HR ≥ 80 HPM was used to collect three cardiac cycles. The 20G cannula was fixed in the superficial vein of the right upper extremity using a dual barrel high-pressure injector. First, inject 50 – 70 ml (320 mg I/ml, 2 ml/kg) of the nonionic contrast agent Youweixian intravenously at a rate of 5 ml/s, and then inject 40 ml of normal saline at the same rate. The entire scanning process was triggered by the surestart software. The monitoring area was centrally located within the thoracic aorta within the extent of the scan. The monitoring area was placed in the thoracic aorta at the central level of the scanning field. The trigger threshold is set to 200 HU.

4. Preprocessing of CCTA images

Due to the fact that the images come from two centers, the acquisition protocol, scanner, and spatial resolution inevitably affected the obtained radiomics features.

Therefore, we first performed a unified pre-processing on all images before extracting features. Image preprocessing can also reduce the risk of overfitting and improve the generalization ability of the models. A software package that performs quantitative analysis (A.K. software, GE Healthcare) was used to preprocess the CCTA images before extracting the radiomics features. Each sequence of the images is resampled to a resolution of 1 x1 x 1 mm^3 through linear interpolation and the gray level of the images needs to be discretized and normalized to 32 orders.

5. **Automatic segmentation of ROI and acquisition of CT-FFR and pFAI**

Arterial phase images of CCTA in each patient were imported in DICOM format into the CQK analysis platform of PHIgo (version 1.5.1, GE Healthcare) software for automated segmentation of the pericoronary adipose tissue (PCAT) around coronary artery and whole myocardium. Firstly, PCAT around the stenosis lesion on coronary segments (≥2 mm) can be accurately delineated according to the 18-segment guidelines on the arterial phase images. Secondly, a simulated three-dimensional myocardial visualization image can be obtained through the segmentation and reconstruction of the main coronary segments of coronary segments for the extraction of whole myocardial tissue. Thirdly, Radiologists A and B evaluated images of all patients for semi-automatic segmentation of PCAT-ROI and myocardial-ROI, and manually corrected images with poor segmentation results. The manual correction of images includes the following important steps: (1) Remove myocardial fifibrous fifilaments; (2) Remove non-cardiomyocardial tissue; (3) Correct segmentation errors of coronary

artery segments and three-dimensional myocardium; (4) Correct the ROI segmentation range of PCAT and the whole myocardial tissue in detail. For PCAT-ROI, radiologists A and B independently corrected 121 and 138 patients, respectively. For myocardial ROI, radiologists A and B corrected 51 and 60 patients, respectively. After the above steps were processed, the CT-FFR and pFAI at the target lesion were calculated using semi-automatic software. The detailed process is shown in Figure 2E, F. Then, the radiomics features extraction in pericoronary adipose tissue ROI (PCAT-ROI) and the myocardium ROI (myocardium-ROI) was performed based on an open source package of Python (Pyradiomics). Finally, the repeatability of features extracted from inter observers was evaluated using ICCs. To eliminate the central effects of two centers, we used ComBat to normalize and gather the data distributions (Figure S2).

6. The specific calculation process CT-FFR

CT-fractional flow reserve (CT-FFR) is a new marker that can be obtained from CCTA images using fluid dynamics technology to simulate invasive FFR (1, 2). With invasive FFR as the reference standard, CT-FFR has shown high sensitivity in predicting the hemodynamic significance of coronary artery stenosis compared with studies using CCTA alone (3, 4). Therefore, we used the PHIGo workstation (Precision health institution, Version 1.5.1) to measure the CT-FFR of target lesion. The specific calculation process of CT-FFR is based on two hypotheses: 1) The CCTA imaging could be regarded as the static arterial first-pass imaging of coronary artery; 2) The venous ICM concentration Cv(t) is close to zero. At least 5ml/s of contrast agent shall

be injected to optimize the enhancement intensity during the first-pass arterial phase. Subsequently, it enters the early part of the first circulation of non - ionic contrast agent, at which point the contrast agent rarely enter the vein. At this point, we believe that the venous ICM concentration $C_V(t)$ is close to zero. We record the concentration as $fC_a(t)$ during the arterial input and $fC_V(t)$ during the venous output. The calculation formula and example diagram are as follows:

Fick principle: $$\frac{dct(t)}{dt} = ft*Ca(t) - ft*Cv(t)$$

$$ft = \left[\frac{dCt(t)}{dt} \right] / \left[Ca(t) \right]$$

$$FFR = \frac{f\,stenosis}{f\,aorta}$$

The ICM attenuates X-rays directly proportionally to iodine content in tissue.

$$FFR = \frac{\frac{dC_{stenosis}(t)}{dt}}{\frac{dC_{aorta}(t)}{dt}} \Rightarrow FFR = \frac{CT\ value_{stenosis}}{CT\ value_{aortic\ reference\ point}}$$

The above was the calculation process of CT-FFR. In order to display the calculation of CT-FFR more vividly, the specific process was detailed in Figure 2E.

7. The formula of Elastic Net Regression and display of optimal signatures.

(1) The formula of Elastic Net Regression

$$Cost(W) = \sum_{i=1}^{N} \left(y_i - W^T x_i \right)^2 + \lambda \alpha \|W\|_1 + \frac{\lambda(1-\alpha)}{2} \|W\|_2^2$$

(2) Myocardium signatures

original_glcm_Imc2 wavelet.LLH_glcm_MCC

wavelet.HHL_glcm_Correlation

wavelet.HHL_glszm_LargeAreaHighGrayLevelEmphasis

wavelet.HHH_glszm_GrayLevelNonUniformity

wavelet.LLL_glszm_GrayLevelVariance

(3) PCATsignatures

original_shape_Sphericity

log.sigma.3.0.mm.3D_glrlm_LongRunLowGrayLevelEmphasis

log.sigma.3.0.mm.3D_glszm_LargeAreaLowGrayLevelEmphasis

wavelet.LHL_ngtdm_Strength

wavelet.LHH_firstorder_Median

wavelet.HLL_firstorder_Mean

wavelet.HLH_firstorder_Median

wavelet.HHH_firstorder_Mean

wavelet.HHH_glcm_DifferenceVariance

8. Decision curve analysis (DCA)

In our study, DCA method was used to evaluate the dimensionality reduction of data by calculate the range of threshold probabilities in which a prediction model was clinically useful. The concept of DCA can be illustrated by the equation below:

$$\frac{a - c}{d - b} = \frac{1 - P_t}{P_t}$$

where d – b represents the influence of unnecessary dimension reduction. If the dimension reduction is guided by the prediction model, d – b is the harm related to a false-positive result compared with a true-negative result. On the contrary, a – c represents the result of rejecting beneficial dimension reduction, in another way, the harm from a false-negative result compared with a true-positive result. Pt represents

where the expected benefit of dimension reduction as same as the expected benefit of refraining from treatment.

References

1. Min JK, Taylor CA, Achenbach S, et al. Noninvasive Fractional Flow Reserve Derived From Coronary CT Angiography: Clinical Data and Scientific Principles. *JACC Cardiovasc Imaging*. (2015) 8:1209-1222. doi: 10.1016/j.jcmg.2015.08.006.

2. Zhuang B, Wang S, Zhao S, et al. Computed tomography angiography-derived fractional flow reserve (CT-FFR) for the detection of myocardial ischemia with invasive fractional flow reserve as reference: systematic review and meta-analysis. *Eur Radiol*. (2020) 30:712-725. doi: 10.1007/s00330-019-06470-8

3. Fujimoto S, Kawasaki T, Kumamaru KK, et al. Diagnostic performance of on-site computed CT-fractional flow reserve based on fluid structure interactions: comparison with invasive fractional flow reserve and instantaneous wave-free ratio. *Eur Heart J Cardiovasc Imaging*. (2019) 20: 343-352. doi:10.1093/ehjci/jey104.

4. Ihdayhid AR, Sakaguchi T, Linde JJ, Sørgaard MH, Kofoed KF, Fujisawa Y, et al. Performance of computed tomography-derived fractional flow reserve using reduced-order modelling and static computed tomography stress myocardial perfusion imaging for detection of haemodynamically significant coronary stenosis. *Eur Heart J Cardiovasc Imaging*. (2018) 19:1234-1243. doi: 10.1093/ehjci/jey114.

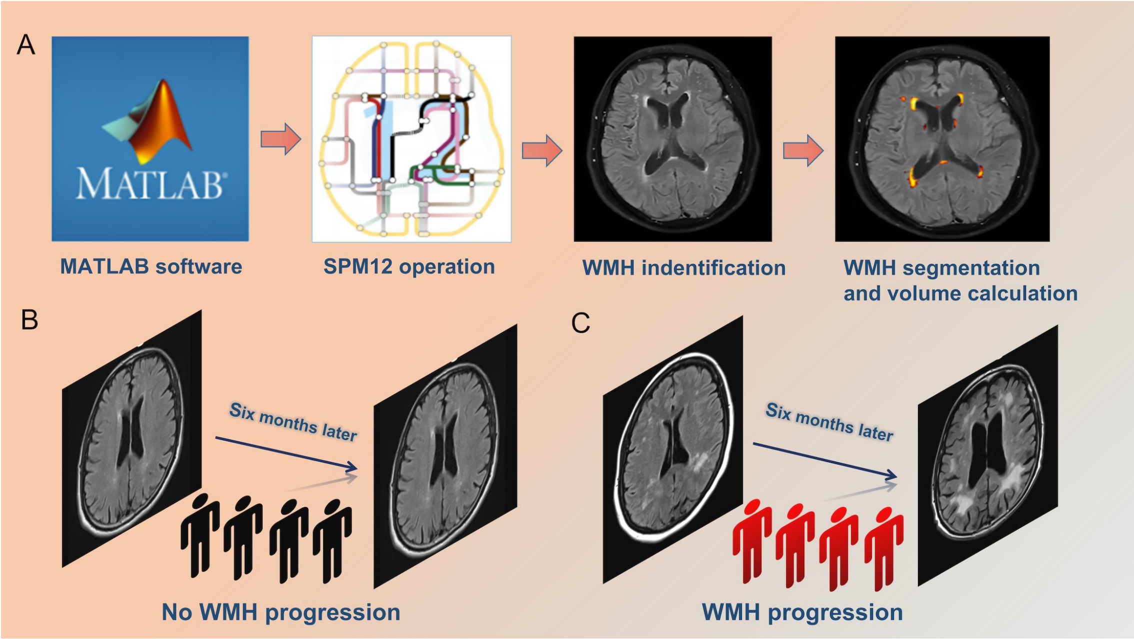

FIGURE S1

Automated segmentation of WMH.

(A) Example diagram of automatic software segmentation WMH process. (B, C) The schematic diagrams of two consecutive brain MRI images of the WMH progression and no -WMH progression groups.

WMH, white matter hyperintensity

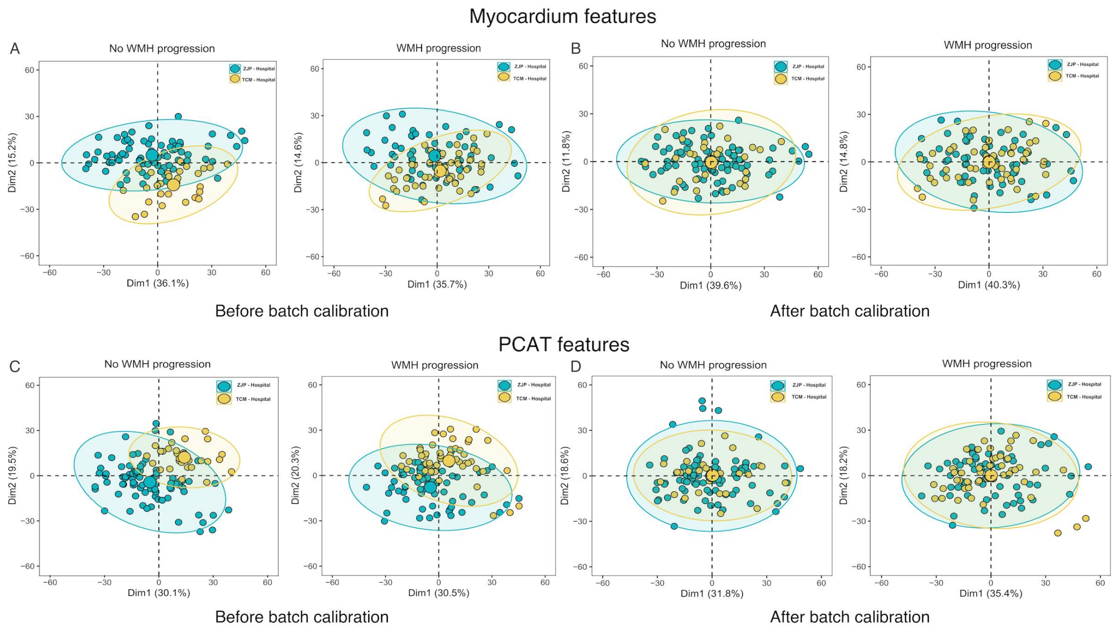

FIGURE S2

Principal Component Analysis (PCA) and refining of features for the prediction models. **(A, B)** The main components of important myocardium radiomics features before and after using Combat were visualized in the two-dimensional scattering map. **(C, D)** The main components of important PCAT radiomics features before and after using Combat were visualized.

PCAT, pericoronary adipose tissue; WMH, white matter hyperintensity

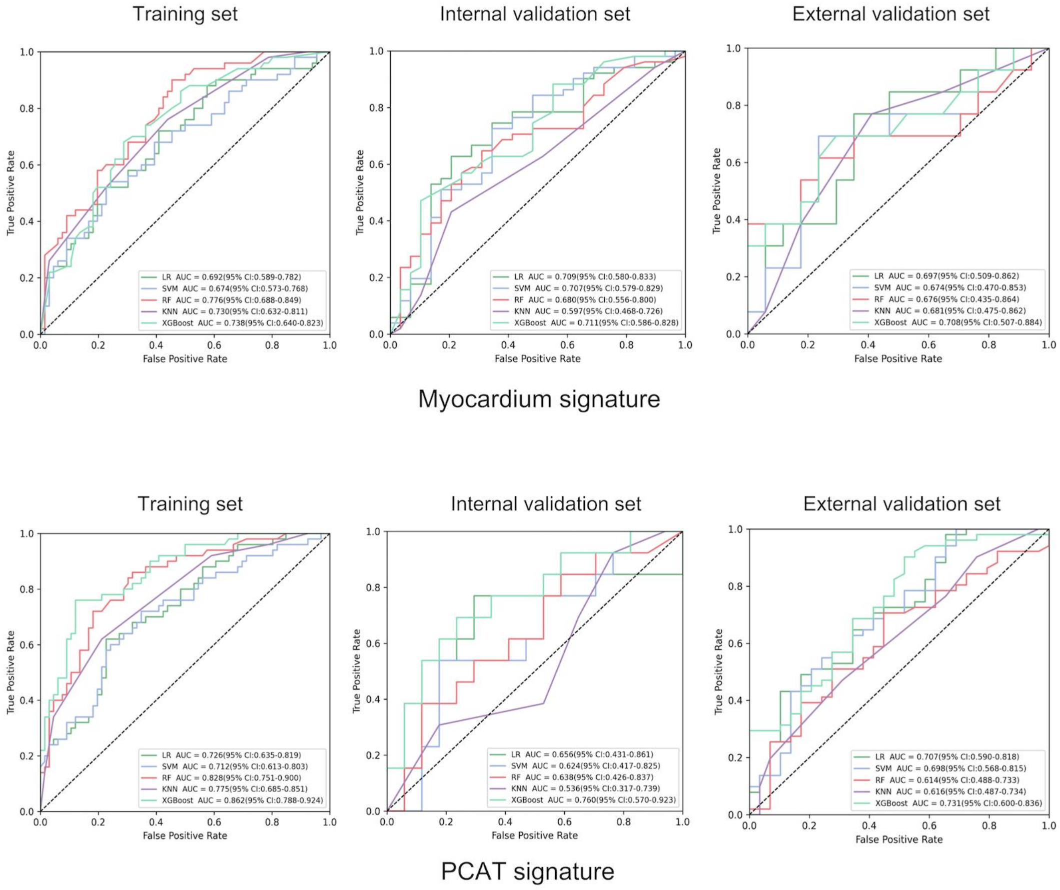

FIGURE S3

Diagnostic performance of myocardium and PCAT radiomics signatures in predicting WMH progression using different algorithms.

(A, B, C) ROC curves for myocardium radiomics signatures for prediction of WMH progression in the training set, internal validation set and external validation set. **(D, E, F)**ROC curves for PCAT radiomics signatures for prediction of WMH progression in the training set, internal validation set and external validation set.

LR, Logistic Regression; SVM, Support Vector Machine; RF, Random Forest; KNN, k-nearest neighbor; XGBoost, eXtreme Gradient Gradient Boosting Machine

Table S1 Clinical and demographic characteristics of all enrolled patients in ZJP Hospital and TCM Hospital

Characteristic	ZJP Hospital				TCM Hospital			
	Study Population (n=146)	Progression of WMH		*P*	Study Population (n=80)	Progression of WMH		*P*
		No（n=83）	Yes (n=63)			No（n=29）	Yes (n=51)	
Demographics								
Age(y)[a]	70.6 ± 6.40	69.9 ± 6.4	71.6 ± 6.3	0.107	73.7 ± 11.4	70.9 ± 13.0	75.3 ± 10.2	0.094
Male sex(n)[b]	98 (67.1%)	53 (63.9%)	45 (71.4%)	0.335	42 (52.5%)	14 (48.3%)	28 (54.9%)	0.568
Cardiovascular risk factors								
BMI(kg/m^2)[a]	23.5 ± 3.66	23.8 ± 3.3	23.0 ± 4.0	0.175	23.2 ± 3.5	23.9 ± 3.1	22.9 ± 3.7	0.214
Hypertension(n)[b]	95 (65.1%)	50 (60.2%)	45 (71.4%)	0.160	52 (65%)	20 (69.0%)	32 (62.7%)	0.575
Diabetes mellitus(n)[b]	49 (33.6%)	25 (17.1%)	24 (38.1%)	0.312	32 (40%)	13 (44.8%)	19 (37.2%)	0.506
Hyperlipidemia(n)[b]	13 (8.9%)	9 (10.8%)	4 (6.3%)	0.345	21 (26.3%)	12 (41.4%)	9 (17.6%)	0.020
Smoking in past 5 years(n)[b]	55 (37.7%)	27 (32.5%)	28 (44.4%)	0.141	14 (17.5%)	5 (17.2%)	9 (17.6%)	0.963
Alcohol intake in past 5 years(n)[b]	41 (28.1%)	21 (25.3%)	20 (31.7%)	0.391	12 (15.0%)	4 (13.8%)	8（15.7%)	0.820
Stenotic vessels number[b]				0.123				0.114
1	55 (37.7%)	37 (44.6%)	18（28.6%)		27 (33.8%)	11 (37.9%)	16 (31.4%)	
2	43 (29.5%)	23 (27.7%)	20（31.7%)		25 (31.3%)	5 (17.2%)	20 (39.2%)	
3	48 (32.9%)	23 (27.7%)	25（39.7%)		28 (35.0%)	13 (44.8%)	15 (29.4%)	
Stenosis classification[b]				0.732				0.559
1	74 (50.7%)	44 (53.0%)	30 (47.6%)		30 (37.5%)	13 (44.8%)	17 (33.3%)	

2	46 (31.5%)	24 (28.9%)	22 (34.9%)		33 (41.3%)	10 (34.5%)	23 (45.1%)	
3	26 (17.8%)	15 (18.1%)	11 (17.5%)		17 (21.3%)	6 (20.7%)	11 (21.6%)	
pFAI[a]	-66.4 ± 6.6	-69.1 ± 5.7	-62.8 ± 5.9	<0.001*	-71.7 ± 5.8	-74.0 ± 4.3	-70.4 ± 6.2	0.007*
CT-FFR[a]	0.7 ± 0.1	0.8 ± 0.1	0.7 ± 0.1	0.007*	0.8 ± 0.1	0.8 ± 0.1	0.7 ± 0.1	0.028*
The volume of WMH at baseline[a]	7.2 ± 8.3	4.5 ± 6.9	10.9 ± 8.6	<0.001*	9.3 ± 7.1	8.5 ± 7.2	9.8 ± 7.0	0.404
The volume of WMH at follow-up[a]	8.1 ± 8.8	3.9 ± 5.6	13.6 ± 9.2	<0.001*	11.2 ± 8.1	6.4 ± 5.6	14.1 ± 8.0	<0.001*
MR interval (months)[a]	14 ± 5	14 ± 5	14 ± 4	0.576	14 ± 5	13 ± 5	14 ± 5	0.658

[a] Presented as mean ± standard deviation, and Student's t test was performed to compare these variables; [b] Presented as frequencies and percentages, and Chi-square test was used for the comparisons of these variables.

BMI, body mass index; pFAI, pericoronary fat attenuation index; CT-FFR, CT fractional flow reserve; WMH, white matter hyperintensity

Bold values represent $p < 0.05$, with significant statistical differences.

Table S2 The diagnostic performance of myocardium and PCAT radiomics signatures by different algorithms

	Algorithms	AUC (95% CI)	Accuracy	Sensitivity	Specificity
**Myocardium**					
Training	LR	0.69 (0.59 - 0.78)	0.65	0.72	0.59
	SVM	0.67 (0.57 - 0.77)	0.66	0.54	0.76
	RF	0.78 (0.69 - 0.85)	0.70	0.90	0.55
	KNN	0.73 (0.63 - 0.81)	0.65	0.76	0.56
	**XGBoost**	**0.74 (0.64 - 0.82)**	**0.70**	**0.68**	**0.71**
Internal validation	LR	0.70 (0.51 - 0.86)	0.63	0.770	0.53
	SVM	0.67 (0.47 - 0.85)	0.63	0.46	0.77
	RF	0.68 (0.44 - 0.86)	0.50	0.69	0.35
	KNN	0.68 (0.48 - 0.86)	0.67	0.770	0.59
	**XGBoost**	**0.71 (0.51 - 0.88)**	**0.70**	**0.69**	**0.71**
External validation	LR	0.71 (0.58 - 0.83)	0.71	0.78	0.59
	SVM	0.71 (0.58 - 0.83)	0.60	0.47	0.83
	RF	0.68 (0.56 - 0.80)	0.65	0.69	0.59
	KNN	0.60 (0.47 - 0.73)	0.58	0.63	0.48
	**XGBoost**	**0.71 (0.59 - 0.83)**	**0.64**	**0.63**	**0.66**
**PCAT**					
Training	LR	0.73 (0.64 - 0.82)	0.71	0.62	0.77
	SVM	0.71 (0.61 - 0.80)	0.68	0.72	0.65
	RF	0.83 (0.75 - 0.90)	0.76	0.86	0.68

	KNN	0.78 (0.69 - 0.85)	0.72	0.62	0.79
	**XGBoost**	**0.86 (0.79 - 0.92)**	**0.83**	**0.76**	**0.88**
Internal validation	LR	0.66 (0.43 - 0.86)	0.70	0.54	0.82
	SVM	0.62 (0.42 - 0.83)	0.63	0.54	0.71
	RF	0.64 (0.43 - 0.84)	0.57	0.62	0.53
	KNN	0.54 (0.32 - 0.74)	0.43	0.39	0.47
	**XGBoost**	**0.76 (0.57 - 0.92)**	**0.70**	**0.54**	**0.82**
External validation	LR	0.71 (0.59 - 0.82)	0.60	0.57	0.66
	SVM	0.70 (0.57 - 0.82)	0.64	0.63	0.66
	RF	0.61 (0.49 - 0.73)	0.63	0.71	0.48
	KNN	0.62 (0.49 - 0.73)	0.55	0.47	0.69
	**XGBoost**	**0.73 (0.60 - 0.84)**	**0.56**	**0.47**	**0.72**

LR, Logistic Regression; SVM, Support Vector Machine; RF, Random Forest; KNN, k-nearest neighbor; XGBoost, eXtreme Gradient Gradient Boosting
